# Diaqua­bis(4-bromo-2-formyl­phenolato-κ^2^ *O*,*O*′)cobalt(II)

**DOI:** 10.1107/S1600536808026068

**Published:** 2008-09-06

**Authors:** Yu Xiao, Min Zhang

**Affiliations:** a The Guangxi Key Laboratory of Environmental Engineering, Protection and Assessment (Department of Resources and Environmental Engineering, Guilin University of Technology), Guilin 541004, People’s Republic of China

## Abstract

In the title complex, [Co(C_7_H_4_BrO_2_)_2_(H_2_O)_2_], the Co^II^ ion, which lies on a crystallographic inversion center, is coordin­ated by four O atoms from two bidentate 4-bromo-2-formyl­phenolate ligands and two O atoms from two water ligands in a slightly distorted octa­hedral environment. In the crystal structure, one-dimensional chains are formed through inter­molecular O—H⋯O hydrogen bonds, which are further linked into a two-dimensional network through Br⋯Br inter­actions [Br⋯Br = 3.772 (4) Å].

## Related literature

For related literature, see: Cohen *et al.* (1964 ▶); Desiraju (1989 ▶); Mathews & Manohar (1991 ▶); Willey *et al.* (1994 ▶); Zaman *et al.* (2004 ▶); Zhang *et al.* (2007 ▶); Zordan *et al.* (2005 ▶); Chen *et al.* (2008 ▶).
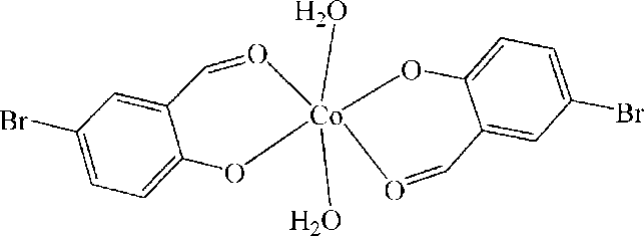


## Experimental

### 

#### Crystal data

[Co(C_7_H_4_BrO_2_)_2_(H_2_O)_2_]
*M* *_r_* = 494.99
Monoclinic, 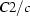

*a* = 29.527 (5) Å
*b* = 4.7406 (8) Å
*c* = 11.6314 (18) Å
β = 103.162 (3)°
*V* = 1585.3 (4) Å^3^
*Z* = 4
Mo *K*α radiation
μ = 6.15 mm^−1^
*T* = 293 (2) K
0.21 × 0.19 × 0.19 mm

#### Data collection

Bruker SMART-CCD diffractometer
Absorption correction: none
3884 measured reflections
1553 independent reflections
1290 reflections with *I* > 2σ(*I*)
*R*_int_ = 0.033

#### Refinement

*R*[*F* ^2^ > 2σ(*F* ^2^)] = 0.040
*wR*(*F* ^2^) = 0.095
*S* = 1.04
1553 reflections
106 parameters
H-atom parameters constrained
Δρ_max_ = 0.55 e Å^−3^
Δρ_min_ = −0.32 e Å^−3^

### 

Data collection: *SMART* (Bruker, 2004 ▶); cell refinement: *SAINT* (Bruker, 2004 ▶); data reduction: *SAINT*; program(s) used to solve structure: *SHELXS97* (Sheldrick, 2008 ▶); program(s) used to refine structure: *SHELXL97* (Sheldrick, 2008 ▶); molecular graphics: *ORTEP-3* (Farrugia, 1997 ▶) and *ORTEPIII* (Burnett & Johnson, 1996 ▶); software used to prepare material for publication: *SHELXTL* (Sheldrick, 2008 ▶).

## Supplementary Material

Crystal structure: contains datablocks global, I. DOI: 10.1107/S1600536808026068/lh2677sup1.cif

Structure factors: contains datablocks I. DOI: 10.1107/S1600536808026068/lh2677Isup2.hkl

Additional supplementary materials:  crystallographic information; 3D view; checkCIF report

## Figures and Tables

**Table:** 

Co1—O2	2.013 (2)
Co1—O1	2.099 (2)
Co1—O3	2.149 (3)

**Table:** 

O2^i^—Co1—O2	180
O2—Co1—O1	87.86 (10)
O2—Co1—O1^i^	92.14 (10)
O1—Co1—O1^i^	180
O2—Co1—O3^i^	90.20 (10)
O1—Co1—O3^i^	86.83 (10)
O2—Co1—O3	89.80 (10)
O1—Co1—O3	93.17 (10)
O3^i^—Co1—O3	180

Symmetry code: (i) 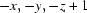.

**Table 2:** Hydrogen-bond geometry (Å, °)

*D*—H⋯*A*	*D*—H	H⋯*A*	*D*⋯*A*	*D*—H⋯*A*
O3—H3⋯O1^ii^	0.85	2.12	2.842 (4)	142
O3—H3*B*⋯O2^iii^	0.85	1.93	2.725 (4)	155

Symmetry codes: (ii) 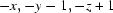; (iii) 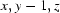.

**Fig. 1.**
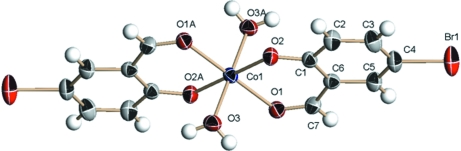
A view of (I), showing 30% probability displacement ellipsoids [symmetry code: (A) -x, -y, -z+1]

**Fig. 2.**
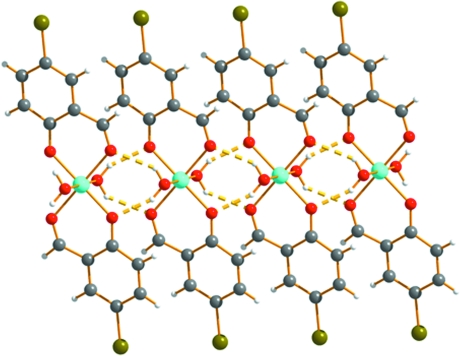
1-D chain of (I). Dashed lines indicate hydrogen bonds.

**Fig. 3.**
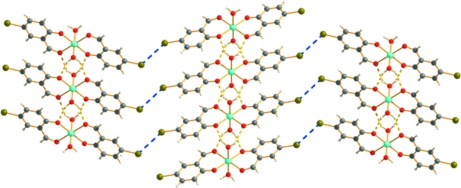
2-D structure of (I). Blue dashed lines indicate Br..Br interactions and yellow dashed lnies show hydrogen bonds.

## supplementary crystallographic information

### Comment

Halogens have a ubiquitous presence in both inorganic and organic chemistry. Schiff bases of bromo substituents on aromatic groups have aroused increasing interest in recent years because these halogenated compounds are an attractive target for use in supramolecular chemistry and crystal engineering wherein the halogen atoms are directly involved in forming intermolecular interactions (Cohen *et al.*, 1964, Zordan *et al.*, 2005; Desiraju, *et al.* 1989, Zaman *et al.*, 2004; Zhang, *et al.*, 2007, Chen, *et al.*, 2008). The title compound, (I), contains the bromo ligand 5-bromo-2-hydroxy-benzaldehyde, with one Br atom accessible at the periphery of each ligand.

In the molecular structure of (I), the Co^II^ ion is coordinated by four O atoms from two bidentate 5-bromo-2-hydroxy-benzaldehyde ligands and two O atoms from two H_2_O ligands forming a slightly distorted octahedral geometry (Fig. 1). In the crystal structure, 1-D chains are  formed through O–H···O hydrogen bonds (O3···O1^i^,   2.842 (4)Å; O3···O2^ii^, 2.725 (4); symmetry codes: (i)-x, -y-1, -z+1; (ii)  x, y-1, z). Each molecule of (I) forms eight hydrogen bonds, four of which are donor hydrogen bonds and four are acceptor hydrogen bonds. The 1-D chains are further linked into a 2-D network *via* Br1···Br1 interactions. The shortest Br1···Br1 distance is 3.772 Å, (Mathews & Manohar, 1991; Willey *et al.*, 1994) observed between Br1 and Br1^iii^,   Br1 and Br1^iv^ [symmetry codes: (iii) 1/2-x,-1/2+y,1/2-z; (iv) 1/2-x,1/2+y,1/2-z] .

### Experimental

Distilled water (30 ml) containing 5-bromo-2-hydroxy-benzaldehyde (0.201 g, 1 mmol) was dropwise added to an aqueous solution containing amino-methanesulfonic acid (0.111 g, 1 mmol) and sodium hydroxide (0.040 g, 1 mmol) with stirred during 10 min. After stirring for 1 h, an aqueous solution of cobalt chloride (0.237 g, 1 mmol) was added to the resulting solution and stirred for 2 h and filtrate. the filtration was left to stand at room temperature. After 12 days, red crystals were produced from the filtrate (yield: 76.4 %, based on Co).

### Refinement

H atoms were positioned geometrically and were treated as riding atoms, with C–H distances of 0.93 Å and *U*_iso_(H) = 1.2 *U*_eq_(C), and with and O–H distance of 0.85 Å and *U*_iso_(H) = 1.5 *U*_eq_(O) .

### Crystal data

**Table:**

[Co(C_7_H_4_BrO_2_)_2_(H_2_O)_2_]	*F*(000) = 964
*M**_r_* = 494.99	*D*_x_ = 2.074 Mg m^−^^3^
Monoclinic, *C*2/*c*	Mo *K*α radiation, λ = 0.71073 Å
Hall symbol: -C 2yc	Cell parameters from 3884 reflections
*a* = 29.527 (5) Å	θ = 2.8–26.0°
*b* = 4.7406 (8) Å	µ = 6.15 mm^−^^1^
*c* = 11.6314 (18) Å	*T* = 293 K
β = 103.162 (3)°	Prism, red
*V* = 1585.3 (4) Å^3^	0.21 × 0.19 × 0.19 mm
*Z* = 4	

### Data collection

**Table:**

Bruker SMART-CCD diffractometer	1290 reflections with *I* > 2σ(*I*)
Radiation source: fine-focus sealed tube	*R*_int_ = 0.033
graphite	θ_max_ = 26.0°, θ_min_ = 2.8°
φ and ω scans	*h* = −27→36
3884 measured reflections	*k* = −5→5
1553 independent reflections	*l* = −13→14

### Refinement

**Table:**

Refinement on *F*^2^	Primary atom site location: structure-invariant direct methods
Least-squares matrix: full	Secondary atom site location: difference Fourier map
*R*[*F*^2^ > 2σ(*F*^2^)] = 0.040	Hydrogen site location: inferred from neighbouring sites
*wR*(*F*^2^) = 0.095	H-atom parameters constrained
*S* = 1.04	*w* = 1/[σ^2^(*F*_o_^2^) + (0.0409*P*)^2^ + 2.4257*P*] where *P* = (*F*_o_^2^ + 2*F*_c_^2^)/3
1553 reflections	(Δ/σ)_max_ < 0.001
106 parameters	Δρ_max_ = 0.55 e Å^−^^3^
0 restraints	Δρ_min_ = −0.32 e Å^−^^3^

### Special details

**Table:**

Geometry. All esds (except the esd in the dihedral angle between two l.s. planes) are estimated using the full covariance matrix. The cell esds are taken into account individually in the estimation of esds in distances, angles and torsion angles; correlations between esds in cell parameters are only used when they are defined by crystal symmetry. An approximate (isotropic) treatment of cell esds is used for estimating esds involving l.s. planes.
Refinement. Refinement of F^2^ against ALL reflections. The weighted R-factor wR and goodness of fit S are based on F^2^, conventional R-factors R are based on F, with F set to zero for negative F^2^. The threshold expression of F^2^ > 2sigma(F^2^) is used only for calculating R-factors(gt) etc. and is not relevant to the choice of reflections for refinement. R-factors based on F^2^ are statistically about twice as large as those based on F, and R- factors based on ALL data will be even larger.

### Fractional atomic coordinates and isotropic or equivalent isotropic displacement parameters (Å^2^)

**Table:**

	*x*	*y*	*z*	*U*_iso_*/*U*_eq_	
Co1	0.0000	0.0000	0.5000	0.0288 (2)	
Br1	0.222905 (17)	0.41219 (14)	0.34123 (5)	0.0652 (2)	
O1	0.03315 (9)	−0.2051 (5)	0.3818 (2)	0.0354 (6)	
O2	0.05893 (9)	0.2221 (5)	0.5574 (2)	0.0333 (6)	
O3	0.02353 (10)	−0.3049 (5)	0.6373 (2)	0.0377 (6)	
H3B	0.0416	−0.4195	0.6135	0.057*	
H3	0.0003	−0.3971	0.6490	0.057*	
C1	0.09460 (13)	0.2474 (8)	0.5103 (3)	0.0309 (8)	
C2	0.13013 (14)	0.4390 (9)	0.5592 (4)	0.0405 (10)	
H2	0.1280	0.5390	0.6266	0.049*	
C3	0.16768 (15)	0.4831 (10)	0.5110 (4)	0.0447 (11)	
H3A	0.1908	0.6094	0.5463	0.054*	
C4	0.17148 (14)	0.3393 (10)	0.4089 (4)	0.0421 (10)	
C5	0.13865 (13)	0.1447 (9)	0.3593 (3)	0.0377 (9)	
H5	0.1419	0.0447	0.2928	0.045*	
C6	0.09989 (13)	0.0949 (8)	0.4087 (3)	0.0304 (8)	
C7	0.06866 (15)	−0.1242 (8)	0.3544 (3)	0.0367 (9)	
H7	0.0763	−0.2157	0.2907	0.044*	

### Atomic displacement parameters (Å^2^)

**Table:**

	*U*^11^	*U*^22^	*U*^33^	*U*^12^	*U*^13^	*U*^23^
Co1	0.0330 (4)	0.0264 (4)	0.0284 (4)	−0.0043 (3)	0.0104 (3)	−0.0022 (3)
Br1	0.0404 (3)	0.1038 (5)	0.0569 (3)	−0.0135 (3)	0.0226 (2)	0.0066 (3)
O1	0.0411 (16)	0.0322 (14)	0.0362 (15)	−0.0047 (12)	0.0157 (12)	−0.0056 (11)
O2	0.0323 (15)	0.0353 (15)	0.0340 (14)	−0.0067 (12)	0.0114 (12)	−0.0080 (11)
O3	0.0474 (17)	0.0312 (14)	0.0364 (15)	−0.0001 (12)	0.0138 (13)	−0.0008 (11)
C1	0.031 (2)	0.032 (2)	0.030 (2)	0.0007 (16)	0.0078 (16)	0.0034 (15)
C2	0.037 (2)	0.049 (3)	0.036 (2)	−0.0060 (19)	0.0098 (19)	−0.0067 (18)
C3	0.035 (2)	0.054 (3)	0.043 (3)	−0.012 (2)	0.006 (2)	0.001 (2)
C4	0.031 (2)	0.056 (3)	0.041 (2)	−0.003 (2)	0.0123 (18)	0.009 (2)
C5	0.037 (2)	0.048 (3)	0.031 (2)	0.0001 (19)	0.0122 (17)	0.0015 (18)
C6	0.034 (2)	0.0287 (19)	0.0287 (19)	0.0008 (16)	0.0062 (16)	0.0003 (15)
C7	0.045 (3)	0.038 (2)	0.032 (2)	0.0022 (19)	0.0177 (18)	−0.0033 (17)

### Geometric parameters (Å, °)

**Table:**

Co1—O2^i^	2.013 (2)	C1—C2	1.406 (6)
Co1—O2	2.013 (2)	C1—C6	1.424 (5)
Co1—O1	2.099 (2)	C2—C3	1.368 (6)
Co1—O1^i^	2.099 (2)	C2—H2	0.9300
Co1—O3^i^	2.149 (3)	C3—C4	1.395 (6)
Co1—O3	2.149 (3)	C3—H3A	0.9300
Br1—C4	1.894 (4)	C4—C5	1.367 (6)
O1—C7	1.225 (5)	C5—C6	1.412 (5)
O2—C1	1.299 (4)	C5—H5	0.9300
O3—H3B	0.8500	C6—C7	1.436 (6)
O3—H3	0.8500	C7—H7	0.9300
			
O2^i^—Co1—O2	180	O2—C1—C6	123.8 (3)
O2^i^—Co1—O1	92.14 (10)	C2—C1—C6	116.8 (3)
O2—Co1—O1	87.86 (10)	C3—C2—C1	122.1 (4)
O2^i^—Co1—O1^i^	87.86 (10)	C3—C2—H2	118.9
O2—Co1—O1^i^	92.14 (10)	C1—C2—H2	118.9
O1—Co1—O1^i^	180	C2—C3—C4	120.3 (4)
O2^i^—Co1—O3^i^	89.80 (10)	C2—C3—H3A	119.9
O2—Co1—O3^i^	90.20 (10)	C4—C3—H3A	119.9
O1—Co1—O3^i^	86.83 (10)	C5—C4—C3	120.1 (4)
O1^i^—Co1—O3^i^	93.17 (10)	C5—C4—Br1	120.4 (3)
O2^i^—Co1—O3	90.20 (10)	C3—C4—Br1	119.5 (3)
O2—Co1—O3	89.80 (10)	C4—C5—C6	120.3 (4)
O1—Co1—O3	93.17 (10)	C4—C5—H5	119.9
O1^i^—Co1—O3	86.83 (10)	C6—C5—H5	119.9
O3^i^—Co1—O3	180	C5—C6—C1	120.3 (3)
C7—O1—Co1	125.4 (2)	C5—C6—C7	116.2 (3)
C1—O2—Co1	129.1 (2)	C1—C6—C7	123.5 (3)
Co1—O3—H3B	107.9	O1—C7—C6	127.9 (4)
Co1—O3—H3	109.2	O1—C7—H7	116.1
H3B—O3—H3	108.2	C6—C7—H7	116.1
O2—C1—C2	119.4 (3)		

Symmetry codes: (i) −*x*, −*y*, −*z*+1.

### Hydrogen-bond geometry (Å, °)

**Table:**

*D*—H···*A*	*D*—H	H···*A*	*D*···*A*	*D*—H···*A*
O3—H3···O1^ii^	0.85	2.12	2.842 (4)	142.
O3—H3B···O2^iii^	0.85	1.93	2.725 (4)	155.

Symmetry codes: (ii) −*x*, −*y*−1, −*z*+1; (iii) *x*, *y*−1, *z*.
